# Exploring pathogenesis, prevalence, and genetic associations in Chiari malformation type 1: a contemporary perspective

**DOI:** 10.2478/abm-2024-0021

**Published:** 2024-09-20

**Authors:** Siti Nornazihah Mohd Rosdi, Suzuanhafizan Omar, Mazira Mohamad Ghazali, Ab Rahman Izaini Ghani, Abdul Aziz Mohamed Yusoff

**Affiliations:** Department of Neurosciences, School of Medical Sciences, University Sains Malaysia, Health Campus, Kubang Kerian 16150, Kelantan, Malaysia

**Keywords:** Chiari malformation type 1, environmental factors, genetic associations, pathogenesis, treatment options

## Abstract

Chiari malformation type 1 (CM 1) entails a structural defect in the cerebellum, involving the herniation of cerebellar tonsils toward the foramen magnum. The symptomatic or asymptomatic nature of CM 1 is contingent upon the condition of malformation in the spinal cord. This review presents an updated perspective on the prevalence of CM 1, its pathogenesis, genetic associations, and treatment. CM 1 exhibits a higher prevalence in adult females than males. Despite the incomplete understanding of the exact cause of CM 1, recent research suggests the involvement of both genetic and environmental factors in its development. One of the reasons for the occurrence of CM 1 in individuals is the smaller posterior cranial fossa, which manifests as typical morphological features. Additionally, environmental factors can potentially interact with genetic factors, modifying the observable characteristics of the disease and affecting the symptoms, severity, and development of the condition. Notably, headaches, neck pain, dizziness, and neurological deficits may be exhibited by individuals with CM 1, highlighting the importance of early diagnosis. Magnetic resonance imaging (MRI) serves as an alternative diagnostic technique for monitoring the symptoms of CM 1. Multiple genetic factors are likely to contribute to a cascade of abnormalities in CM 1. Early studies provided evidence, including clustering within families, bone development, and co-segregation with known genetic syndromes, establishing CM 1’s association with a genetic basis. Furthermore, surgery is the only available treatment option to alleviate symptoms or hinder the progression of damage to the central nervous system (CNS) in CM 1 cases.

## What is CM 1?

Chiari malformation type 1 (CM 1) represents an abnormality within the cerebellum, which is marked by the downward herniation of the cerebellar tonsils through the foramen magnum (FM), measuring at least 5 mm, without encompassing the brain stem according to the McRae’s line [1]. This herniation causes compression of neural tissue at the craniovertebral junction, potentially leading to neurological issues, possibly linked to syringomyelia or hydrocephalus. Although many CM 1 cases are asymptomatic, symptoms may vary depending on tissue compression, nerve impingement, and accumulation of cerebrospinal fluid (CSF) in the spinal cord [2].

In prenatal diagnosis, medical practitioners use magnetic resonance imaging (MRI) or ultrasonography to evaluate disease severity and treatment requirements. While some CM 1 cases have been linked to trauma, many are congenital in nature [3]. Hence, researchers have suggested that typical CM 1 arises due to inadequate paraxial mesoderm development, affecting occipital bone sclerotomes and leading to a smaller, shallower posterior fossa (PF) incapable of accommodating the normal cerebellar size [4].

The occurrence of CM 1 among numerous family members, rather than solely in monozygotic twins, strongly implies that the malformation is genetically based, even in the absence of Mendelian inheritance [5]. A study conducted in 2014 indicated that the exact genetic cause associated with CM 1 had yet to be determined due to limited research, despite numerous studies indicating the genetic contribution to CM 1 based on twin studies, family connections, and co-occurrence with known genetic syndromes [6]. Despite data collected regarding the link between genetic variations and the presence of CM 1 in both isolated and syndromic cases, a comprehensive genome-wide investigation in healthy parents has not been carried out.

## History of CM 1

A variety of conditions, marked by diverse causes, pathophysiological mechanisms, and clinical manifestations, constitute Chiari malformations. Despite their diverse origins, these conditions have in common anatomical abnormalities that impact the brain stem and cerebellum. These conditions are classified within the category of craniospinal malformations [7].

Being named after Viennese pathologist Hans Chiari, CM 1 was discovered in 1891. In his documentation, a 17-year-old woman who succumbed to typhoid fever and experienced hydrocephalus was described. Interestingly, no symptoms related to the cerebellum or medulla were exhibited by the patient. The malformation was characterized as a “peg-like” elongation of the cerebellar tonsils and medial divisions of the inferior lobes of the cerebellum into cone-shaped projections, which extend along with the medulla into the spinal canal, while the medulla remained unaffected [8].

In 1894, German pathologist Julius Arnold provided a pathological post-mortem account of an infant exhibiting the herniation of cerebellar tonsils and the fourth ventricle through the FM, while leaving the medulla unaffected. Additionally, the infant was diagnosed with spina bifida [8]. The resemblance between Arnold’s findings and those of Hans Chiari led to the coining of the term “Arnold-Chiari malformation,” which is now commonly used in medical terminology to refer to type 2 Chiari malformation.

## Prevalence of CM 1 and world perspective

CM 1 is not limited to specific countries but it has a global impact. CM 1, the most prevalent type, affects approximately 1 in every 1000 births [9]. Utilizing advanced diagnostic technology for CM 1, extensive data have been gathered regarding CM 1 prevalence in various countries, considering factors such as ethnicity, race, and geographical location. In the United States, a study revealed a higher predominance of CM 1 in adult females compared to males. This finding is based on a cohort study of 364 patients with CM 1 within the U.S. population, where 76% were female and 24% were male, with an average age of onset 24.9 ± 15.8 years [10]. This discovery aligns with the observations of Arnautovic et al. [11], who, based on surgical series, reported a higher prevalence of females over males among adults (57% and 34%, respectively). In pediatric series, the distribution was more even, with 28% girls and 25% boys.

In Japan, the incidence of CM 1 with tonsillar ectopia measuring 1–4 mm below the FM was found to be 0.24%, accounting for 12 out of 5,000 cases. Conversely, in the study involving U.S. population, 14% of 200 subjects had CM 1 with cerebellar tonsils herniating 1–3 mm below the FM. In a study involving 600 European-American subjects, the occurrence of CM 1 was observed in only 5%, showing the herniation of cerebellar tonsils over 5 mm below the FM. This suggests that tonsillar ectopia with a range of 1–5 mm is more frequent in the Euro-American population compared to Japan, an Asian country [12].

In terms of ethnicity, a study was carried out on the population in northern New Zealand in 2001, which comprises 11.7% Pacific people, 12.5% Maori, and 75.5% Caucasians or other ethnicities. The results revealed that among 100,000 individuals, Pacific Islanders (18.4%) and Maori (15.4%) have a higher incidence of syringomyelia linked to CM 1 compared to Caucasians or individuals of other ethnicities (5.4%). However, the underlying reasons for these ethnic disparities remain undisclosed and continue to be subjects of investigation [13].

### Pathogenesis of CM 1

The exact cause of Chiari malformation, particularly CM 1, remains incompletely understood. However, recent research suggests that both genetic and environmental factors play a role in the development of CM 1. The primary characteristic of CM 1 is a structural irregularity in the cerebellum, resulting in the positioning of cerebellar tonsils below the foreman magnum.

### Congenital factors

#### Abnormal skull development (smaller posterior cranial fossa)

CM 1 is frequently recognized as a congenital condition that appears at birth. Primarily, abnormalities in the skull base are also observed in most cases of CM 1 [14]. Typical morphological features identified in individuals with CM 1 include a reduced size of the PF, the area at the skull’s base, compared to what is typically observed. This limited space results in the cerebellum, or more specifically, the cerebellar tonsils, descending toward the spinal canal. The previous discovery corresponds to the investigation conducted by Stovner et al. [15], revealing X-ray measurements of patients’ skull dimensions from a lateral perspective. Their study involved 33 individuals with CM 1 and 40 individuals without this condition. Consequently, patients diagnosed with CM 1 displayed a reduction in the size and depth of their posterior cranial fossa (PCF) when compared to individuals without the condition. This discrepancy emphasized that the smaller the area, the more the cerebellar tonsils extended into the incorrect area of the brain, suggesting that insufficient development in the rear part of the skull might result in the herniation of the hindbrain.

Additionally, CM 1 arises due to congestion in the posterior area, caused by underdeveloped occipital hypoplasia in the fossa, leading to the herniation of the cerebellar tonsils through the FM. Primarily, individuals with CM 1 exhibit abnormalities in the posterior occipital bone, presenting volumetric reduction. This condition is linked to the inadequate development of occipital somites, originating from the paraxial mesoderm during nervous system development [16]. The craniovertebral junction and basicranium are primarily shaped by sclerotome cells originating from the C1 and C2 somites. Previous research and laboratory experiments conducted by Marín-Padilla [17] have supported the theory of a mesodermal origin. Marín-Padilla’s [17] study involved observing occipital hypoplasia, a reduced PF, and a shortened basichondrocranium, which led to the downward displacement of the cerebellum and compression of the medulla. These effects were induced by the administration of large doses of vitamin A, a substance known to impact mesodermal development in gestating hamsters.

### Environmental factors

CM 1 is a complex condition with an inheritance pattern that does not fit within Mendelian inheritance due to its multifaceted nature. It exhibits pleiotropic effects that significantly contribute to the risk of the disease through various genetic variants. Alongside genetic influence, environmental factors also play a role in the development of CM 1. However, the precise understanding of these factors remains incomplete, potentially involving maternal nutrition, exposure to toxins, or other factors impacting fetal development. Moreover, environmental factors can potentially interact with genetic factors that modulate the observable characteristics of the disease, influencing the symptoms, severity, and progression of the condition [18].

For example, an underdeveloped neural tube can result from inadequate maternal nutrition, such as insufficient folic acid intake during pregnancy. Folic acid, which is essential for DNA synthesis and repair, plays a crucial role. However, a deficiency in folic acid can worsen genetic predispositions to CM 1 by hindering the proper development of the fetal skull and brain [19]. Additionally, an experiment conducted by Burren et al. [20] using splotch embryos in a mouse model demonstrated that inducing folate deficiency resulted in a significant increase in cranial neural tube defects. These findings suggest a gene–environment interaction between the loss of *Pax3* function and folate status.

Exposure to teratogens such as drugs, chemicals, alcohol, or toxic substances during pregnancy can elevate the risk of congenital malformations. These substances have the potential to disrupt cellular processes and fetal development, potentially leading to abnormal brain and skull formation, which predisposes the fetus to CM 1. In a previous study performed by Kong et al. [21], a mutation in the *Mosmo* gene was identified, which affects the penetrance, expressivity, and tissue specificity of birth defects induced by exogenous teratogens that inhibit Smoothened (SMO) activity. This research illustrates that gene–environment interactions, which alter ligand sensitivity on the Hedgehog pathway, can impact the penetrance of birth defects.

In a recent 2020 study, it was also mentioned that environmental factors like smoking, physical activities, and nutrition might influence epigenetic changes. This was observed in a case involving monozygotic twins, where differential regulation of 5-methylcytosine DNA and histone acetylation was noticed [22]. The discovery concurs with the investigation carried out by Fraga et al. [23], which observed distinctive epigenetic variations in 5-methylcytosine DNA and histone acetylation in a significant group of monozygotic twins, despite their shared genotype [23].

### CSF dynamics

Baisden [24] suggested that a fundamental issue in CM 1 arises due to the hindrance of CSF flow, resulting in the herniation of the cerebellar tonsils below the FM. The main objective in managing Chiari disease symptoms is to restore typical CSF flow at the craniocervical junction and deter the advancement of syringomyelia.

CSF is a transparent liquid that envelops both the brain and spinal cord, serving as a protective cushion to shield the brain from the skull’s impact [25]. In usual circumstances, CSF circulates unrestricted within the brain and spinal canal. However, when the skull is undersized or malformed, it can result in the downward displacement of the cerebellar tonsils below the FM, potentially giving rise to the formation of a syrinx, which is a fluid-filled cyst within the spinal cord, or syringomyelia [26].

## Genetic syndromes associated with CM 1

Individuals with CM 1 might encounter various symptoms like headaches, neck pain, dizziness, and neurological deficits. Surgery to decompress the FM has been a common approach for treating Chiari malformation, but it carries certain risks. Consequently, in the future, alternative approaches such as genetic analysis will be employed in diagnostics rather than MRI, serving as an alternative diagnostic tool to surgery in monitoring the risks of CM 1 [27]. CM 1 was historically regarded as a sporadic condition. However, research involving families has demonstrated that genetic changes contribute to the development of CM 1. The identification of the specific genes or genetic variations responsible for this pathogenic process has the potential to improve the precision of diagnosis and treatment [28]. Unlike many complex medical conditions, CM 1 is believed to result from a series of abnormalities linked to multiple genetic factors. Earlier studies revealed evidence of clustering within families, twin studies, and co-occurrence with recognized genetic syndromes, supporting the idea that CM 1 has a genetic foundation [16]. A summary of previously published reports on gene variants associated with CM1 is provided in **Table 1**.

**Table 1.** List of candidate genes associated with CM 1

**Country**	**Genes**	**Location**	**Function/effects of mutation**	**Region of mutation involved**	**Author**
China	*ERF*	Chromosome 19	Multiple suture = 45.45% of the cases	NM_006494.4(ERF): c.787C>T (p. Gln263Ter)	Wang et al. ^[51]^
			Sagittal suture closure = 20%		
Russia	*MYBPC1*	Chromosome 12 (12q23.2)	Affected individuals have less severe muscle contractions and restricted growth at the base of the skull.	NM_002465.4(MYBPC1): c.517G>A (p. Asp173Asn)	Musolf et al. ^[30]^
	*COX20*	Chromosome 1 (1q43-44)	Microcephaly, structural disorders of the brain and abnormalities of hands/feet.	NM_198076.6(COX20): c.353A>G (p. Asn118Ser)	
USA	*CHD 3*	Chromosome 17	Macrocephaly and development disorder	NM_001005273.3(CHD3): c.50G>A (p. Cys17Tyr); c.70C>T (p. Arg24Trp) and c.74C>T (p. Ala25Val)	Sadler et al. ^[31]^
	*CHD8*	Chromosome 14	Macrocephaly and autism spectrum disorder	NM_001170629.2(CHD8): c.4414C>T (p. Arg1472Ter); c.4514 G>A (p. Trp1505Ter) and c.2907 + 1G>T	
Italy	*DKK1*	Chromosome 10	Affects a pentapeptide motif (NAIKN) during binding with LRPs in WNT signaling.	NM_012242.4 (DKK1): c.121G>A (p. Ala41Thr)	Merello et al. ^[28]^
Spain	*COL7A1*	Chromosome 3	Downregulated in articular cartilage and subchondral bone where osteoarthritis takes place.	NM_000094.4(COL7A1): c.3605G>A (p. Arg1202His)	Urbizu et al. ^[52]^
Spain	*COL6A5*	Chromosome 3	Affects the bone mineral density	NM_001278298.2(COL6A5): c.6814G>T (p. Glu2272Ter)	Urbizu et al. ^[52]^
Japan	*FGFR2*	Chromosome 10	Causes the Crouzon syndrome	NM_000141.5(FGFR2): c.1205C>G (p. Cys342Trp) and c.1021A>G (p. Tyr281Cys)	Fujisawa et al. ^[53]^
USA	*GDF6*	Chromosome 8	Controls proliferation and cellular differentiation in retina and bone formation. Affects BMP signaling.	NM_001001557.4(GDF6): c.746C>A (p. Ala249Glu)	Markunas et al. ^[16]^
USA	*EPAS1*	Chromosome 2	Spinal dysraphism and abnormal vertebral segmentation	NM_001430.5(EPAS1): c.1589C>T (p. Ala530Val) and c.1588G>A (p. Tyr532Cys)	Rosenblum et al. ^[54]^
Italy	*BMP1*	Chromosome 8	Affects the metalloprotease domain which is important in osteogenesis.	NM_001199.4(BMP1): c.941G>A (p. Arg314His)	Merello et al. ^[28]^
Italy	*LRP4*	Chromosome 11	Affects the LDL receptor class B repeat 7 which is part of third β-propeller domain	NM_002334.4(LRP4): c.2552C>G (p. Thr851Arg)	Merello et al. ^[28]^
Turkish	*OLFML2A*	Chromosome 9	Role in development of brain structures	rs7874348^†^	Avşar et al. ^[55]^
	*SLC4A9*	Chromosome 5	Role in fluid secretion regulation (anion exchanger, especially Cl^−^)	rs6860077 ^†^	

BMP, bone morphogenetic protein; CM 1, Chiari malformation type 1; LDL, low-density lipoprotein; NAIKN, amino acids 40–44; WNT, Wingless and Int-1.

The details regarding detected mutations in this study encompass both novel alterations and previously reported changes.

† NCBI SNP database (https://www.ncbi.nlm.nih.gov/snp/) is a database containing curated variant interpretation hosted by National Centre for Biotechnology Information (accessed on May 27, 2024).

### CM 1 among family members

The role of genetics in multiple family members affected by CM 1 cannot be disregarded, as it has been documented in over 30 publications. These studies encompassed more than 250 familial CM 1 cases, including 9 found in monozygotic twins. Most affected patients exhibited symptoms and presented with at least one accompanying condition such as scoliosis or craniosynostosis [29]. Goel et al. [10] conducted a cohort study, revealing that among 364 patients with CM 1, 43 had at least one close relative also affected by CM 1, and they displayed similar symptoms of syringomyelia. An analysis of the pedigrees from 21 families indicated autosomal dominant inheritance as the mode of transmission.

Furthermore, a study carried out by Musolf et al. [30] revealed that CM 1-affected Russian families exhibited a reduced size of the PF attributed to the involvement of myosin binding protein C1 (*MYBPC1*) and cytochrome c oxidase 20 (*COX20*) as candidate genes responsible for this anomaly. Researchers postulated that although variant changes in *MYBPC1* might lead to less severe muscle contraction, they could impede skull growth. Meanwhile, alterations in *COX20* were linked to mitochondrial complex IV deficiency, resulting in symptoms such as ataxia and muscle hypotonia. The less pronounced expression of this phenotype could manifest as a smaller PF [30].

In 2021, Sadleret al. [31] and Haller and Sadler [32] executed a cohort investigation involving 668 CM 1 patients and 232 family members, using the whole-exome sequencing (WES) method to identify genes linked to CM 1. The study identified variations in the chromodomain helicase DNA binding protein 3 and 8 (*CHD3* and *CHD8*) genes, potentially linked to CM 1 and playing a crucial role in brain development. These genes were implicated in contributing to brain abnormalities, evidenced by patients with CM 1 displaying larger head circumferences than the typical range. Moreover, these genes have associations with other neurodevelopmental conditions, including macrocephaly, which has also been observed in CM 1 patients.

### Bone development and CM 1

The correlation between craniofacial and PCF bone development is predominantly observed in CM 1 patients. Nonetheless, the genes responsible for disrupting this development have not been entirely clarified. PCF bone growth has been associated with the Wingless and Int-1 (WNT) signaling pathway, which plays a pivotal role in cranial bone patterning and development. An experiment conducted by Merello et al. [28] identified two heterozygous missense variants of dickkopf-related protein 1 (*DKK1*) in two Italian families with non-isolated CM 1 [28]. This discovery underscored the role of *DKK1* as an inhibitor of WNT signaling, crucial for preserving the proper structural integrity of the developing cranial PF. In 1998, the overexpression of *DKK1* was observed in *Xenopus* embryos, serving as a head inducer and simultaneously antagonizing WNT signaling. This finding emphasized the essential role of *DKK1* in vertebrate head development [33].

In 2022, Martinez-Gil et al.’s [34] research findings further emphasize the involvement of genes linked to bone development, such as *DKK1* and *COL1A2*, in association with CM 1. Moreover, certain bone diseases, like Paget’s disease, are observed to co-segregate with CM 1. Speer et al. [35] also highlighted that the underlying genes associated with CM 1 have pleiotropic effects, potentially leading to cerebellar tonsil herniation, variations in PF volume, or other anomalies in the skull base [35].

### Correlation of CM 1 with multiple genetic syndromes

#### Craniosynostosis

According to Mekbib et al. [29], over 90 Mendelian syndromes have been linked to CM 1. Previous studies have indicated that the clinical phenotype predominantly observed in CM 1, including craniofacial, vertebral, and neurodevelopmental malformations, suggests that CM 1 may be secondary to genetic syndromes [29]. Despite the common association of CM 1 with the underdevelopment of the occipital bone and a small PF, it is noteworthy that primary bone disorders can also contribute to conditions such as osteopetrosis and fibrous dysplasia in the PF skull [36]. Craniosynostosis is frequently observed in individuals with CM 1, occurring in both syndromic and non-syndromic manifestations. The premature fusion of one or more cranial sutures results in an atypical skull shape, leading to overcrowding of the PF and subsequent hindbrain herniation [37].

In a 2005 study, it was discovered that among 95 patients with syndromic craniosynostosis and CM 1, 70% had Crouzon syndrome, 50% had Pfeiffer syndrome, and 100% had Kleeblattschädel deformity [37]. These syndromes result from genetic alterations in the fibroblast growth factor receptor (*FGFR*) gene. The *FGFR* gene plays a crucial role in various processes, including angiogenesis, wound healing, limb development, mesoderm patterning, neuronal differentiation, and skeletogenesis [38]. Mutations in the *FGFR1* gene lead to Pfeiffer syndrome, whereas *FGFR2* mutations are associated with Apert, Crouzon, Pfeiffer type 2, and Jackson-Weiss syndromes. *FGFR3* mutations result in a specific subtype of Crouzon syndrome linked to acanthosis nigricans. Interestingly, mutations in the same *FGFR* genes can give rise to various phenotypes and syndromes, depending on the specific location of the mutations.

#### Cervical spine

Aside from abnormalities in the PCF, CM 1 has also been associated with Mendelian syndromes involving cervical spine disorders, leading to Klippel-Feil syndrome (KFS) and autosomal dominant spondyloepiphyseal dysplasia tarda (autosomal dominant SEDT). KFS represents a rare skeletal condition characterized primarily by the abnormal fusion or union of two or more bones within the neck’s spinal column (cervical vertebrae). While KFS most commonly affects the C2 and C3 vertebrae, genetic mutations can result in the vertebrae not being adequately separated, causing them to stick together like a single piece of bone [39]. The symptoms exhibited in KFS resemble those seen in CM 1, including scoliosis, headaches, and neck pain. However, the typical physical manifestations of KFS involve a short neck, a low posterior hairline, and restricted movement in the upper cervical spine [40].

In 2014, a study reported that approximately 3%–5% of individuals with Chiari malformation also exhibit KFS, and the relevant gene identified was the growth differentiation factor 6 (*GDF6)* gene [6]. In 1995, researchers had previously identified this gene as a potential candidate linked to the segregation of anterior/cervical fusions within a cohort of families affected by KFS [41]. Subsequently, Tassabehji et al. [42] identified missense mutations in both familial and sporadic cases of KFS. Their experiments on *Xenopus laevis* revealed defects in the anterior axial region, thus providing further support for the earlier study.

Autosomal dominant SEDT is an inherited condition impacting bone development. Its signs and symptoms typically become apparent during puberty, although abnormalities can be detected via X-rays at an early age. Individuals affected by autosomal dominant SEDT often display clinical characteristics such as disproportionate short stature, scoliosis, flattened vertebral bodies, hip dislocation, and diminished joint mobility [43]. The *COL2A1* gene is accountable for this disorder. *COL2A1*, a gene encoding the type II procollagen α-1 chain, plays an essential role as the primary structural protein in cartilage and bone. Mutations in this gene result in inaccurate amino acid substitutions, disrupting the proper formation of immature triple-stranded type II collagen molecules [44].

In summary, KFS and autosomal dominant SEDT share connections with CM 1, even though they are linked with additional craniovertebral abnormalities such as basilar invagination, atlanto-occipital assimilation, atlanto-axial instability, and atlas hypoplasia. According to expert opinions, some theories propose that craniovertebral anomalies may contribute to the constriction of neural structures and disrupt the flow of CSF, ultimately leading to the manifestation of symptoms associated with CM 1.

## Treatment of CM 1

Diagnosing CM 1 typically involves employing imaging techniques like MRI to observe the cerebellum’s position and identify any accompanying abnormalities. Surgery represents the sole available treatment to alleviate symptoms or impede the progression of damage to the central nervous system (CNS) in CM 1 cases. The most frequently performed surgical intervention for treating CM 1 is posterior fossa decompression (PFD), which aims to increase the space for the cerebellum, relieve pressure on the spinal cord, and contribute to the restoration of the normal flow of CSF [45]. The PFD procedure can also be conducted in conjunction with reconstructive duraplasty (PFDD), either with or without C1 laminectomy [46]. Additionally, a majority of CM 1 patients who underwent decompression surgery exhibited favorable outcomes in relation to their symptoms, with 77% experiencing an improvement in clinical symptoms and 82% experiencing an enhancement in CSF flow [47].

Despite being a commonly employed treatment for patients with CM 1, the PFD technique raises concerns regarding post-surgical outcomes. It has been observed that PFD alone does not lead to postoperative CSF leakage but it is also associated with a high reoperation rate due to inadequate PFD and CSF circulation when compared to PFD with duraplasty (PFDD) [48].

The most recent research in 2023 has revealed that, apart from medical interventions, non-surgical approaches like non-opioid pain management, specifically designed to address and alleviate symptoms arising from neuropathic pain, offer an alternative treatment option for individuals with CM 1 [49]. This option becomes particularly relevant when patients turn to non-surgical and non-opioid interventions after conventional treatments prove ineffective in reducing their symptoms. A 2019 study noted heightened psychological symptoms, such as depression and anxiety, in individuals with CM 1, regardless of whether they had undergone decompression surgery or not [50]. While surgery can decrease overall pain levels, this research emphasized the potential advantages of incorporating additional evidence-based treatments, like acceptance and commitment therapy and similar forms of psychotherapy, to further lessen the negative impacts of CM 1.

## Conclusion

While the precise cause of CM 1 remains incompletely understood, research indicates a complex interaction between genetics and developmental factors in the development of this neurological condition. CM 1 is recognized as a rare disorder that extends its impact beyond specific countries, affecting individuals worldwide. The compiled references and information within this review suggest that not only genetic factors but also elements such as ethnicity, race, and geographical location significantly influence the prevalence of CM 1.

Fascinatingly, various studies have uncovered potential genetic factors, featuring numerous candidate genes implicated in the pathogenesis of CM 1. However, no single gene mutation seems solely responsible for the structural defects in the cerebellum. Variations in genes associated with craniofacial development, connective tissue integrity, and neural tube formation have been correlated with an elevated susceptibility to CM 1.

The diagnosis of CM 1 is typically established through MRI, a commonly employed method by clinicians for pre-treatment assessment. To enhance future diagnostic capabilities, there should be a more in-depth exploration of genetic markers and molecular mechanisms linked to CM 1. Investigating these molecular mechanisms is essential for achieving a comprehensive understanding of CM 1’s etiology, ultimately refining diagnostic precision and opening avenues for future therapeutic interventions. Additionally, since CM 1 can be hereditary, early molecular diagnosis can serve as an effective screening tool among family members who may have a higher potential of developing the disease.
